# Integrated analysis identifies oxidative stress-related lncRNAs associated with progression and prognosis in colorectal cancer

**DOI:** 10.1186/s12859-023-05203-5

**Published:** 2023-03-03

**Authors:** Rui Chen, Jun-Min Wei

**Affiliations:** Department of Oncology, Qilu Hospital, Cheeloo College of Medicine, Shandong University, Jinan, China

**Keywords:** Oxidative stress, LncRNA, Colorectal cancer, Immunotherapy

## Abstract

**Background:**

Colorectal cancer (CRC) is one of the most common cancers in the world. Oxidative stress reactions have been reportedly associated with oncogenesis and tumor progression. By analyzing mRNA expression data and clinical information from The Cancer Genome Atlas (TCGA), we aimed to construct an oxidative stress-related long noncoding RNA (lncRNA) risk model and identify oxidative stress-related biomarkers to improve the prognosis and treatment of CRC.

**Results:**

Differentially expressed oxidative stress-related genes (DEOSGs) and oxidative stress-related lncRNAs were identified by using bioinformatics tools. An oxidative stress-related lncRNA risk model was constructed based on 9 lncRNAs (*AC034213.1, AC008124.1, LINC01836, USP30-AS1, AP003555.1, AC083906.3, AC008494.3, AC009549.1,* and *AP006621.3*) by least absolute shrinkage and selection operator (LASSO) analysis. The patients were then divided into high- and low-risk groups based on the median risk score. The high-risk group had a significantly worse overall survival (OS) (*p* < 0.001). Receiver operating characteristic (ROC) and calibration curves displayed the favorable predictive performance of the risk model. The nomogram successfully quantified the contribution of each metric to survival, and the concordance index and calibration plots demonstrated its excellent predictive capacity. Notably, different risk subgroups showed significant differences in terms of their metabolic activity, mutation landscape, immune microenvironment and drug sensitivity. Specifically, differences in the immune microenvironment implied that CRC patients in certain subgroups might be more responsive to immune checkpoint inhibitors.

**Conclusions:**

Oxidative stress-related lncRNAs can predict the prognosis of CRC patients, which provides new insight for future immunotherapies based on potential oxidative stress targets.

**Supplementary Information:**

The online version contains supplementary material available at 10.1186/s12859-023-05203-5.

## Background

Colorectal cancer (CRC) is the third most common malignancy worldwide accounting for 9% of all cancer cases, and the fourth most common cause of cancer-related deaths [1]. The combination of folinic acid, 5-fluorouracil, oxaliplatin and/or irinotecan are the efficacious chemotherapy drugs for CRC. In addition, the use of monoclonal antibodies against vascular endothelial growth factor and epidermal growth receptor increased the efficiency of chemotherapy regimens and the survival time of CRC patients, but most patients develop resistance to drugs over a longer course of treatment [2]. Immunotherapy is another promising treatment for CRC, and a growing number of immunotherapy drugs are currently being developed, which can potentially increase treatment effectiveness and reduce side effects [3].

Many risk factors, including environmental factors, smoking, alcohol consumption, diet and obesity, are related to the onset and progression of CRC [4]. The cross talk between these known risk factors can lead to oxidative stress and the overproduction of reactive oxygen species (ROS) in cells, which can gradually result in gene mutations and promote tumor initiation [5–7]. It can also promote tumor growth by activating the receptor tyrosine kinase (RTK), phosphatidylinositol 3‐kinase (PI3K)/Akt, and nuclear factor κB (NF‐κB) pathways. In the later stages of carcinogenesis, excessive ROS may counterintuitively lead to apoptosis [6, 8, 9]. Moreover, ROS continually stimulate the proliferation and survival of tumor cells by activating various transcription factors [10]. Another type of mutation associated with oxidative stress in CRC is DNA microsatellite instability (MSI), which is related to incorrect DNA repair during genome replication [9, 11].

In recent years, it has been increasingly recognized that ROS play a multifaceted role in shaping the tumor microenvironment (TME). Many preclinical studies have shown that immune checkpoint therapy (ICT) and chimeric antigen receptor (CAR)-T-cell therapy can both induce oxidative stress in the TME as well as tumor cells; meanwhile, these two therapies are vulnerable to suppression imposed by ROS derived from the surrounding immunosuppressive cells such as regulatory T cells (Tregs) and myeloid-derived suppressor cells (MDSCs). Therefore, therapeutic strategies should be developed to amplify T-cell-induced oxidative stress in cancer cells but inhibit elevated oxidative stress imposed on effector T cells by the TME [12].

Long noncoding RNAs (lncRNAs) are a class of RNA transcripts with a length greater than 200 nucleotides that have diverse biological functions in cells [13, 14]. It has been revealed that lncRNAs play an important role in tumor growth, metastasis and oxidative stress [15, 16]. For example, the lncRNAs GABPB1-AS1 and GABPB1 can regulate oxidative stress during erastin-induced ferroptosis in the hepatocellular carcinoma cell line HepG2, and high expression levels of GABPB1 are positively correlated with poor prognosis in HCC patients, while high levels of GABPB1-AS1 are correlated with improved overall survival (OS) [17]. The XIST lncRNA promotes oxidative stress-induced migration, invasion, and epithelial-to-mesenchymal transition of osteosarcoma cancer cells through the miR-153-SNAI1 axis [18]. However, the treatment potential of oxidative stress-related lncRNAs has not been explored in CRC. Investigation of oxidative stress-related lncRNAs can help us better understand the roles of oxidative stress in CRC development and provide potential markers for prognosis prediction and targets for CRC treatment.

Currently, the most commonly used prognostic prediction system of CRC in clinical practice is the TNM staging system published by the American Joint Committee on Cancer (AJCC). Previous TNM staging systems have shown excellent performance in formulating a reasonable treatment plan according to the stage, evaluating the efficacy objectively, and judging the prognosis correctly. The traditional view is that the higher the stage is, the worse the prognosis; however, studies have found that stage IIIA patients tend to have a better prognosis than some stage II patients [19]. This suggests that traditional TNM staging has limitations in accurate prognosis prediction, and this system still suffers from the following drawbacks:

I. The system incorporates only three indicators and uses a simple linear approach to classify patients, ignoring the objectivity of patient prognosis as a complex nonlinear phenomenon.
II. The system can no longer accurately predict the clinical prognosis of CRC patients due to its inaccurate weighting and complex time-dependent effects.
III. The system does not integrate newly discovered prognostic predictors for improvement, such as clinical information, pathological information, molecular markers and immune status markers of patients.

In this study, we obtained the transcriptome data and clinical information of CRC patients from The Cancer Genome Atlas (TCGA) database. We further screened the differentially expressed oxidative stress-related genes based on the oxidative stress gene set and then constructed a risk model based on these oxidative stress-related lncRNAs. We verified the effectiveness and accuracy of our model using multiple methods. In summary, this study identified multiple oxidative stress-related genes that are related to the prognosis of patients with CRC and established a risk model based on these lncRNAs that can predict prognosis effectively and accurately, providing novel insights into CRC prognosis prediction and treatment. The proposed model has several novelties compared to the previous TNM system as follows:

I. The prognostic model based on oxidative stress shows high diagnostic accuracy, is valuable for clinical translation and was validated using an independent cohort.
II. The model combines TMB, immune status markers and traditional pathological prognostic indicators to more accurately predict the prognosis of CRC patients.
III. The model can guide clinicians to select the appropriate drug therapy by comparing the sensitivity of patients to common anticancer drugs in high-risk and low-risk populations.

## Results

### DEOSG identification and functional enrichment analyses

**Fig. 1:**
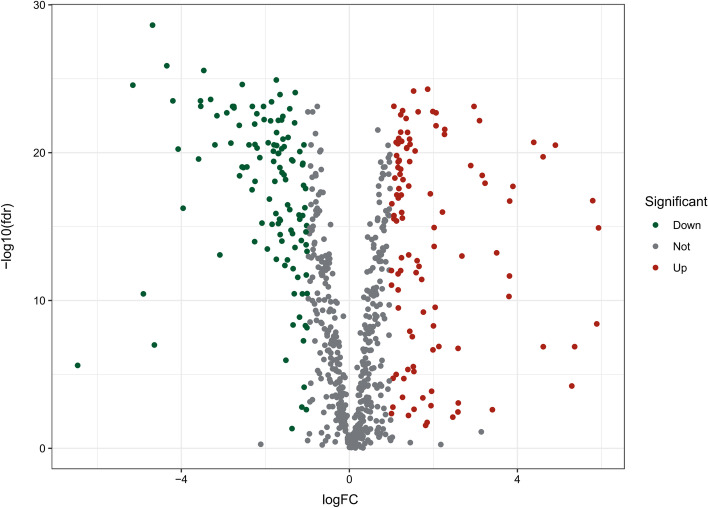
Volcano plot of DEOSGs between CRC and normal samples. Dots in green represent significantly downregulated genes, dots in red represent significantly upregulated genes, and dots in gray represent genes whose expression levels were not changed significantly

**Fig. 2:**
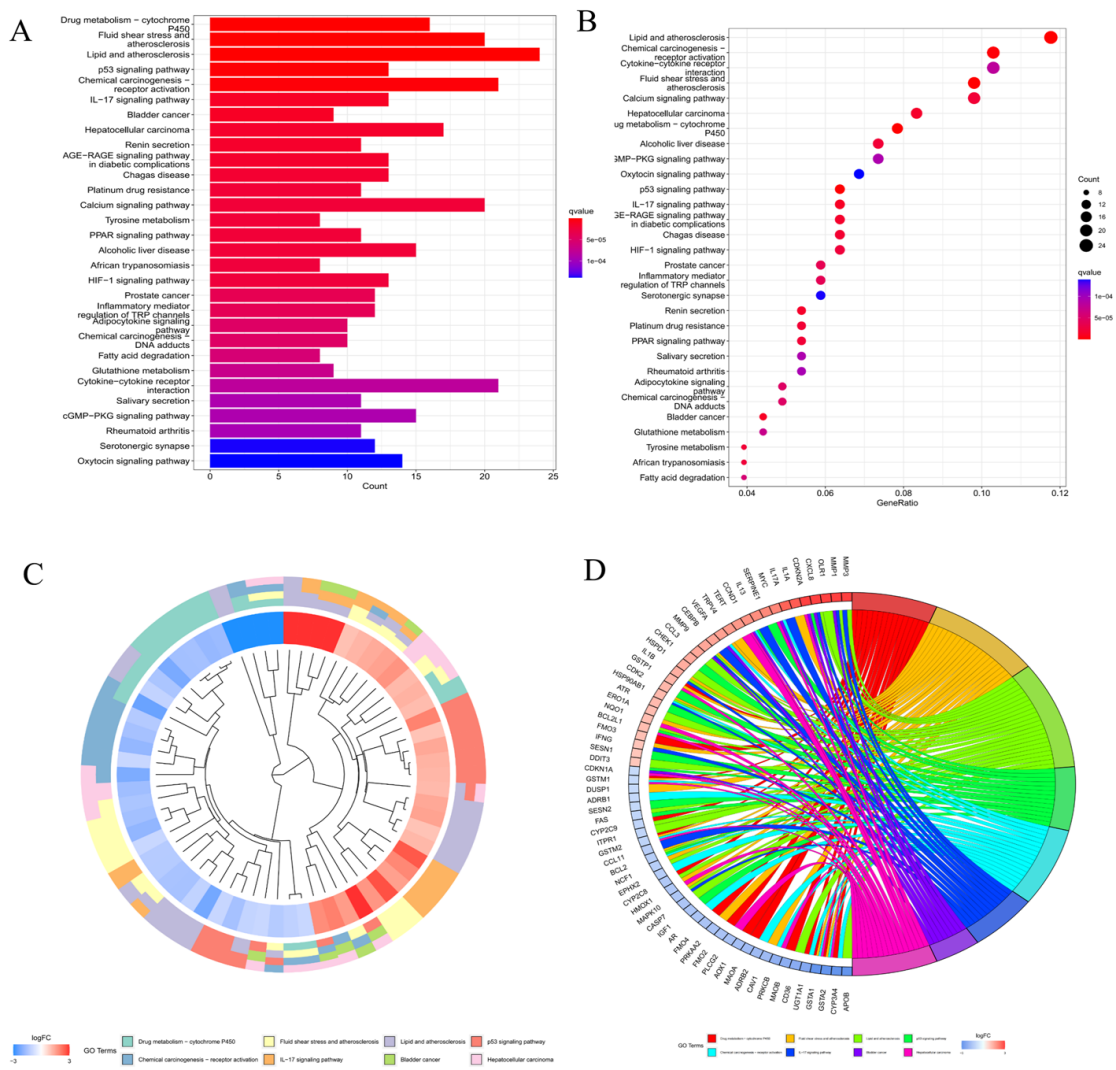
KEGG enrichment analysis of DEOSGs [20–22]. **A**, **B** Top 30 classes of KEGG enrichment terms. Cluster diagram (**C**) and Circle diagram (**D**) showing terms enriched in KEGG analysis

**Fig. 3:**
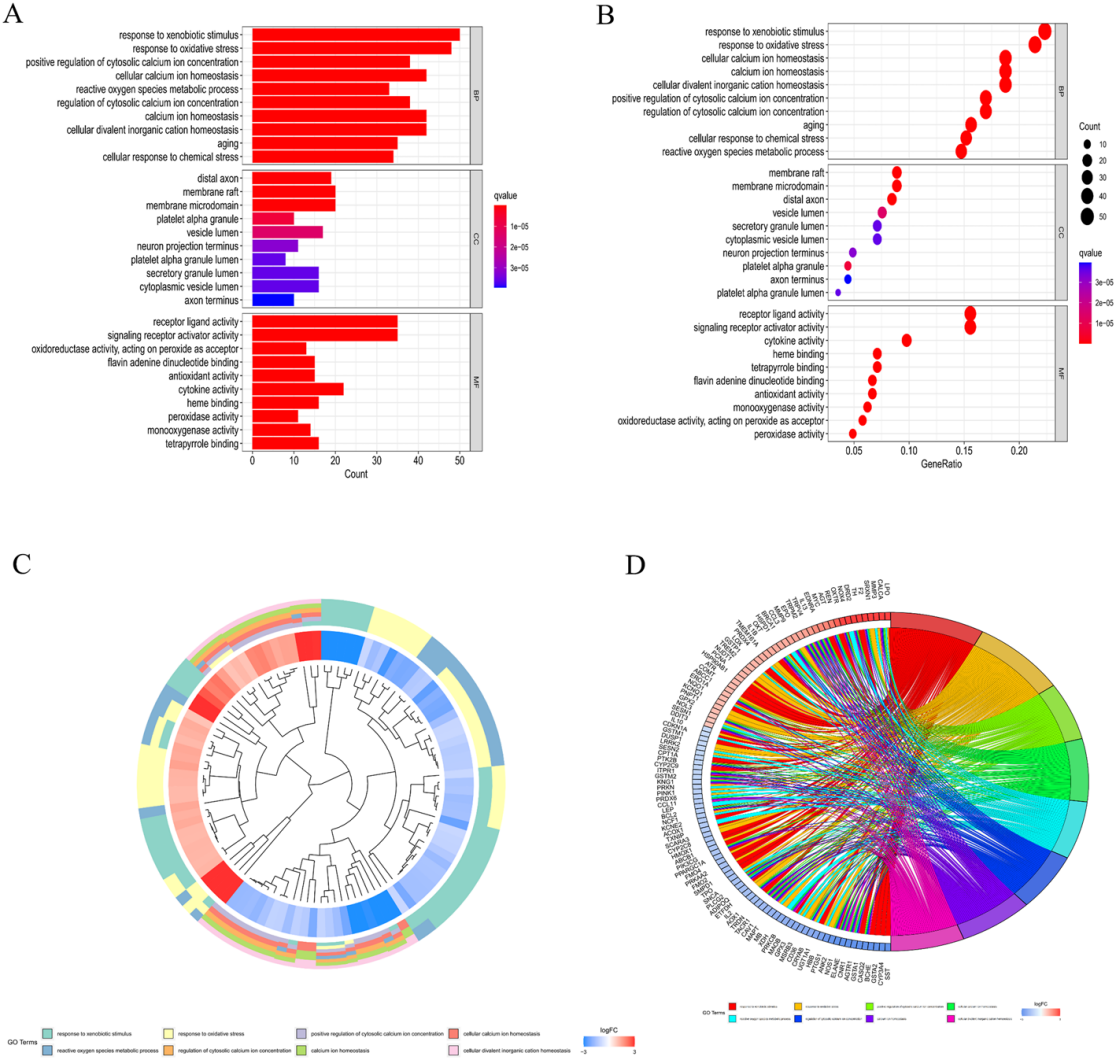
GO enrichment analysis of the DEOSGs. **A**, **B** Top 10 classes of GO enrichment terms in the biological process (BP), cellular component (CC), and molecular function (MF) categories. Cluster diagram (**C**) and circle diagram (**D**) showing terms enriched in GO analysis

A gene list containing 789 oxidative stress-related genes was curated. Differentially expressed genes between 568 CRC samples and 44 adjacent normal tissues were identified. As a result, 226 oxidative stress-related genes, comprising 118 downregulated and 108 upregulated genes, were identified as DEOSGs (*P* < 0.05 and |log2FC|> 1.0). The expression of the DEOSGs in the two subgroups is displayed in Fig. 1. To investigate the potential functional and underlying molecular mechanisms of these DEOSGs, Gene Ontology (GO) and Kyoto Encyclopedia of Genes and Genomes (KEGG) analyses were performed. KEGG analysis showed that the DEOSGs were enriched in the synapse pathway, energy metabolic pathway, drug metabolism pathway, and steroid hormone biosynthesis pathway, all of which are associated with CRC development. The top 10 enriched GO terms included response to oxidative stress, aging, secretory granule lumen, and oxidoreductase activity, acting on peroxide as acceptor (Figs. 2 and 3). All these enrichment analyses indicate that the DEOSGs are involved in metabolism and CRC progression.

### Construction and verification of a prognostic model in patients with CRC

**Fig. 4:**
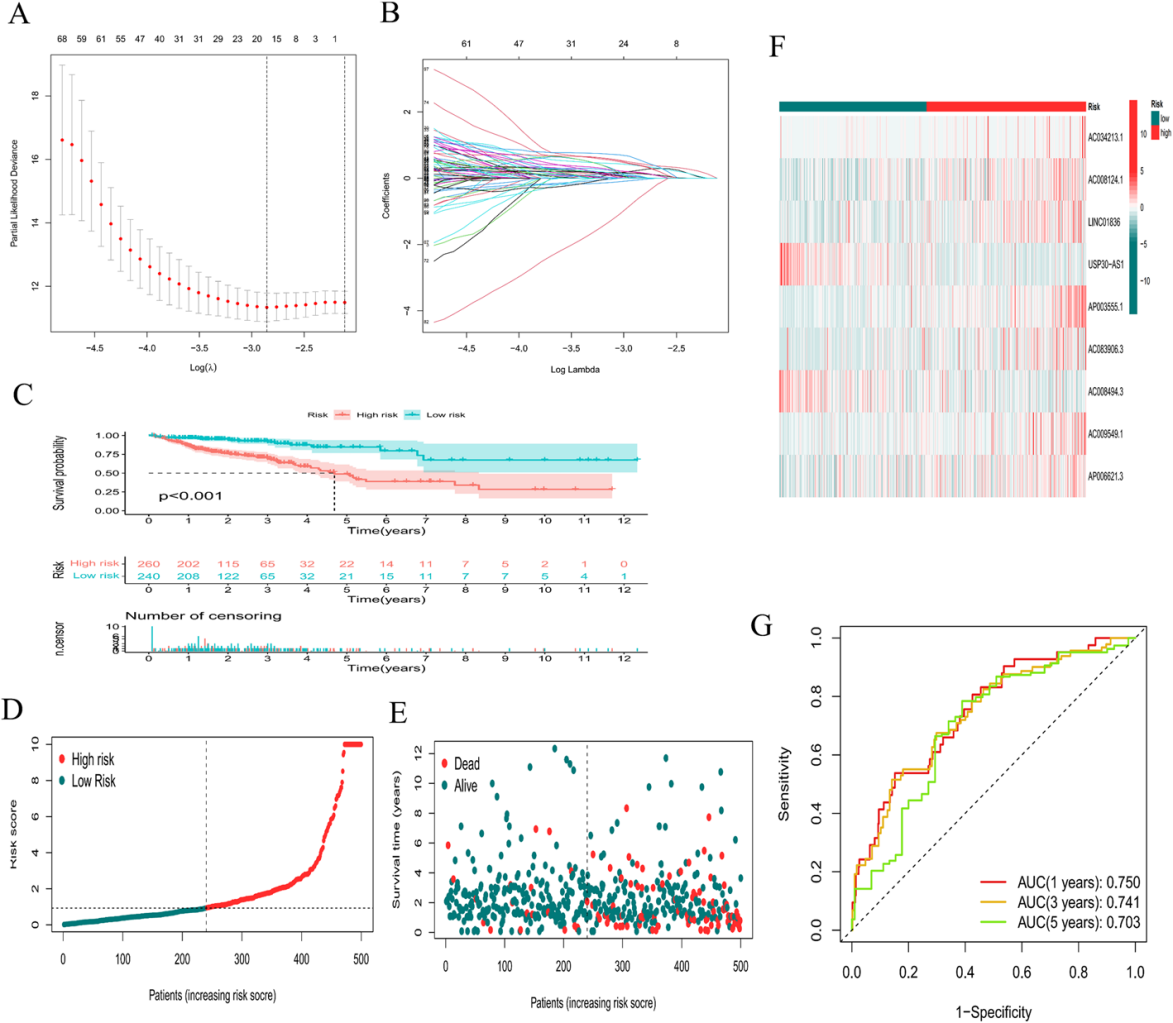
Construction of a prognostic model. **A**, **B** LASSO analysis for determining the number of factors and constructing the prognosis prediction model. **C** Survival curve. **D**, **E** Risk score distribution and survival status **F** heatmap showing the expression levels of the 9 lncRNAs in patients in the low- and high-risk groups. **G** TimeROC curves for forecasting OS

According to univariate Cox regression analysis, we identified prognostic oxidative stress-related lncRNAs significantly correlated with OS (all *P* < 0.05). To avoid overfitting the prognostic model, we performed LASSO regression analysis on these lncRNAs. Finally, 9 lncRNAs (*AC034213.1, AC008124.1, LINC01836, USP30-AS1, AP003555.1, AC083906.3, AC008494.3, AC009549.1,* and *AP006621.3*) were identified that were related to oxidative stress in CRC when the first-rank value of Log(λ) was the minimum likelihood of deviance (Fig. 4A, B). We calculated the risk score with the following formula:

$$\begin{aligned} {\text{Risk score }} & = {\text{ AC}}034213.1{\text{ }} \times {\text{ }}\left( {0.7704} \right){\text{ }} + {\text{ AC}}008124.1{\text{ }} \times {\text{ }}\left( {1.1086} \right) \\ & \quad + {\text{ LINC}}01836 \times {\text{ }}(0.7098){\text{ }} + {\text{ USP}}30 - AS1 \times \left( { - 0.9771} \right){\text{ }} \\ & \quad + {\text{ AP}}003555.1{\text{ }} \times {\text{ }}(1.4298){\text{ }} + {\text{ AC}}083906.3 \times (1.1536) \\ & \quad + {\text{AC}}008494.3 \times {\text{ }}\left( { - 3.6350} \right){\text{ }} + {\text{AC}}009549.1 \times {\text{ }}(1.5010) \\ & \quad + {\text{ AP}}006621.3{\text{ }} \times {\text{ }}(0.4908) \\ \end{aligned}$$

Based on the model, all patients with CRC were separated into low- and high-risk groups according to the median risk score. As shown in Fig. 4C, the OS of patients with CRC significantly decreased as the risk score increased. The number of deaths of CRC patients in the high-risk group was significantly higher than that of patients in the low-risk group (Fig. 4D, E). The heatmap shows the differential expression of 9 lncRNAs between the high- and low-risk groups (Fig. 4F). In addition, time-dependent ROC analysis indicated that the prediction model was quite credible, with the area under the ROC curve (AUC) reaching 0.703 at 5 years, indicating that this prognostic model had moderate specificity and sensitivity (Fig. 4G). This finding suggests that our risk model could predict OS not only for the total population but also for CRC patients.

**Fig. 5:**
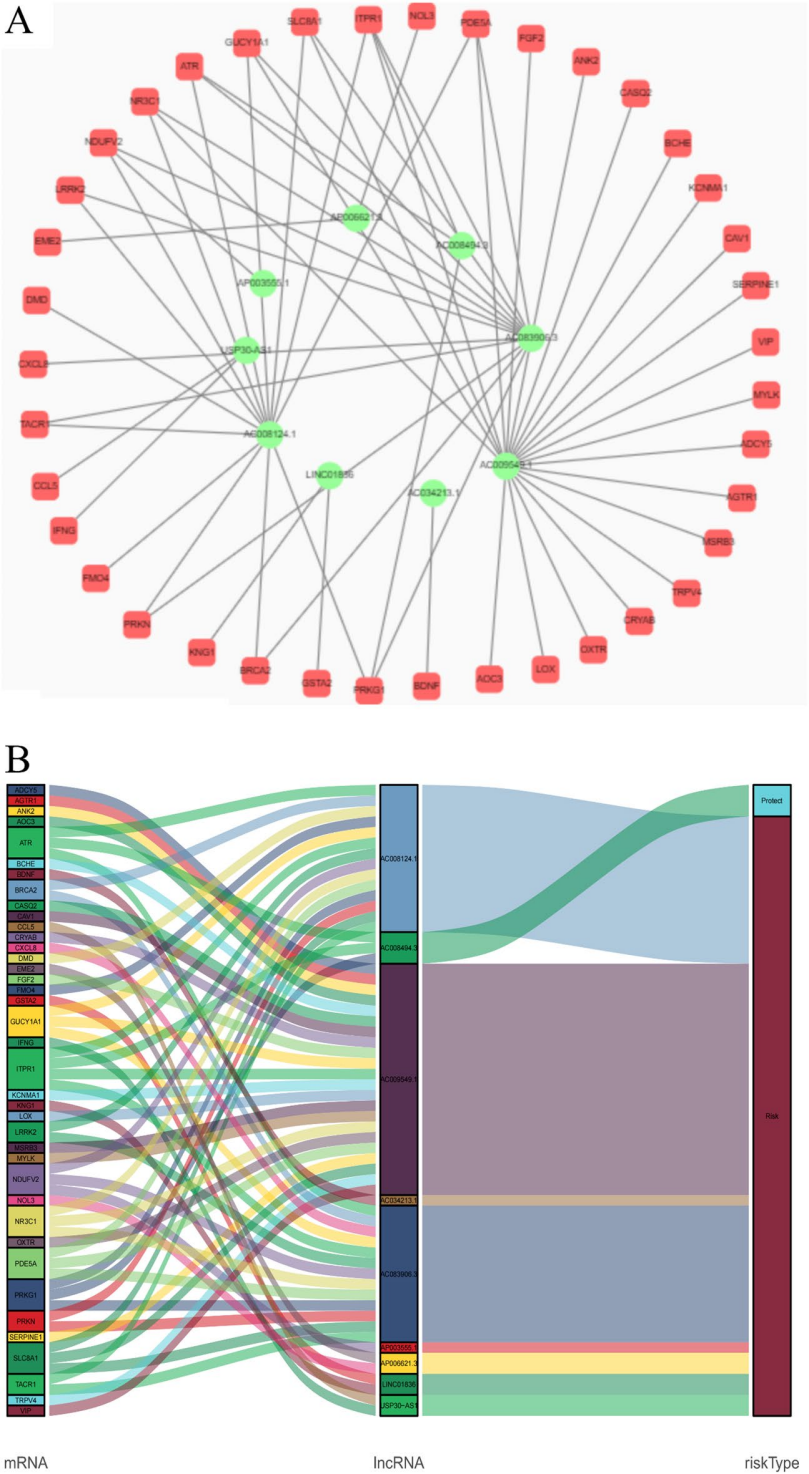
**A** The lncRNA–mRNA network between nine oxidative stress-associated lncRNAs and relevant mRNAs. Green circles indicate oxidative stress-associated lncRNAs. Red squares indicate mRNAs. The line represents a coexpression relationship between the lncRNA and the mRNA. **B** A Sankey diagram showed the co-occurrences of lncRNAs, mRNAs, and characteristics according to the risk score

To better understand the potential effect of lncRNAs on mRNAs in CRC, we built a lncRNA‒mRNA network and used Cytoscape and Sankey diagrams to visualize the network. We constructed the lncRNA‒mRNA coexpression network using the nine screened oxidative stress-associated lncRNAs with Pearson correlation analysis (|R|> 0.4 and *P* < 0.05). A total of 60 lncRNA‒mRNA pairs were filtered, and the correlations among lncRNAs, mRNAs, and the risk score groups were determined by the Sankey diagram (Fig. 5A, B).

**Fig. 6:**
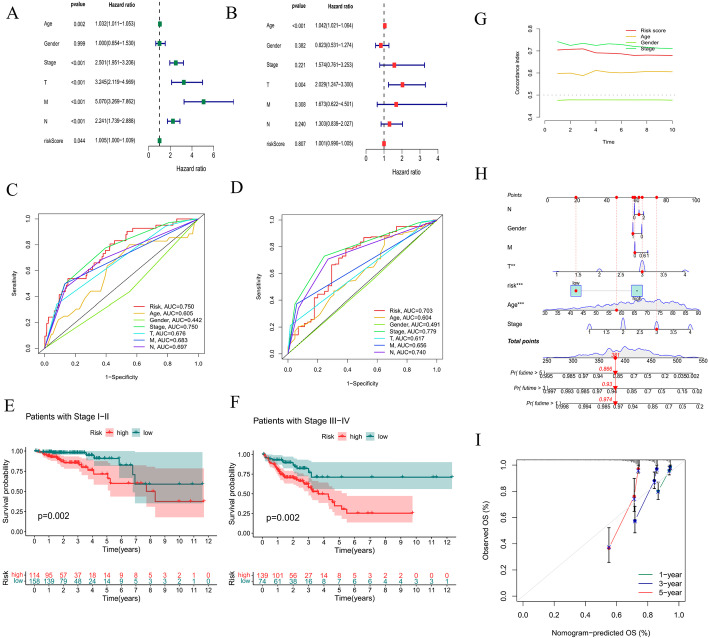
Efficacy evaluation of the constructed prognostic model. Univariate (**A**) and multivariate (**B**) Cox regression analyses of the clinicopathological features. Clinical ROC curves at 1 year (**C**) and 5 years (**D**) for forecasting overall survival. The relationship between the risk score and stage I-II (**E**) and stage III-IV (**F**). **G** The constructed C-index curve. **H** The constructed nomogram of the risk score and other clinical factors for predicting the OS of patients with CRC at 1, 3, and 5 years. **I** The calibration plot of the nomogram

**Fig. 7:**
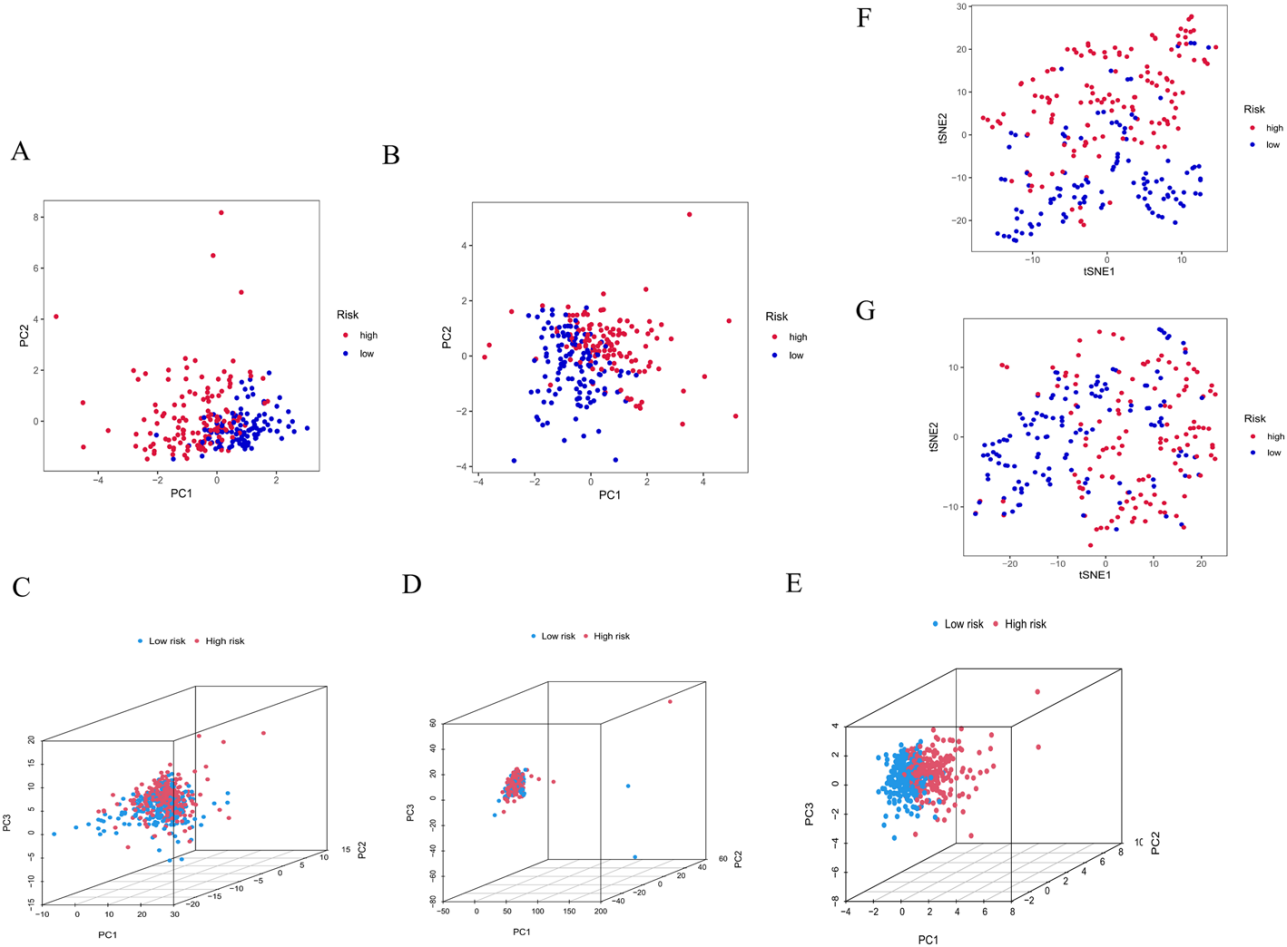
Evaluation of the efficacy of the constructed prognostic model. PCA of the risk groups in the testing (**A**) and training sets (**B**). PCA-3D of the risk groups based on all OS genes (**C**), DEOSGs (**D**), and 9 oxidative stress-related lncRNAs (**E**). The t-SNE of risk groups in the testing (**F**) and training sets (**G**)

Univariate and multivariate Cox regression analyses were performed to determine whether the risk score was an independent prognostic factor. Unfortunately, in univariate Cox regression analysis, although *P* < 0.05, the HR of the risk score was only 1.005; thus, it could not be said that the risk score was an independent prognostic factor yet. The risk score was no longer an independent prognostic factor in multivariate Cox regression analysis (*P* = 0.807) (Fig. 6A, B). The ROC curve at 1 year showed that the prognostic model had better predictive accuracy than other clinical features. The ROC curve at 5 years showed that the prognostic model had better predictive performance than other factors, including age, sex, T stage, N stage, and M stage (Fig. 6C, D). In addition, the conventional clinicopathologic characteristic stage showed the same results (Fig. 6E, F). Additionally, the C-index curve showed that the C-index of the risk score was greater than 0.7, indicating that the model had moderate accuracy (Fig. 6G). A nomogram plot is another quantitative model used to predict the clinical outcomes of patients with CRC. A nomogram plot was developed based on the risk score and other clinical characteristics, allowing the calculation of the survival probabilities of individual patients with CRC at 1, 3, and 5 years (Fig. 6H). The calibration plots indicated good conformity between the predicted and observed outcomes at 3 and 5 years (Fig. 6I). We also employed principal component analyses (PCAs) to demonstrate the distribution patterns of the two subgroups in two-dimensional and three-dimensional graphs (Fig. 7A–E). T-distributed stochastic neighbor embedding (t-SNE) also indicated that two risk groups could be distinguished clearly (Fig. 7F, G). These results indicated that the prognostic model is effective for predicting CRC outcomes and clinical features.

### Evaluation of the prognostic value of 9 oxidative stress-related lncRNAs in an independent gene expression omnibus (GEO) validation cohort

**Fig. 8:**
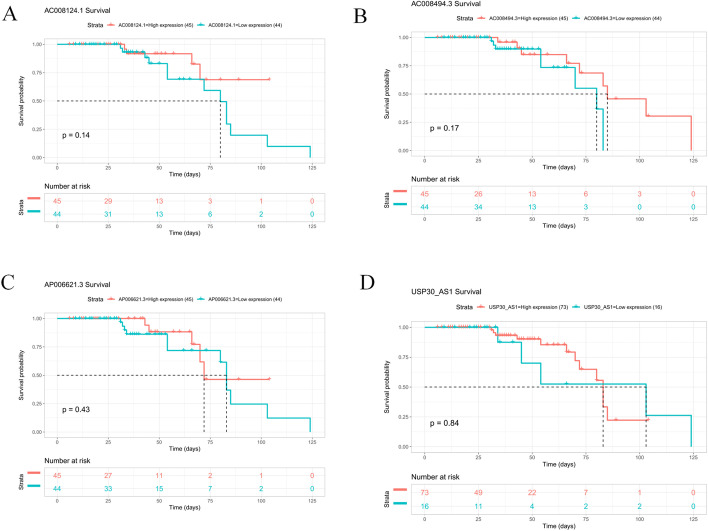
K-M survival curves for 4 target lncRNAs in CRC. The horizontal coordinate of the KM-plot is the survival time and the vertical coordinate is the survival rate. The starting point is the time of the start of follow-up, the falling curve represents patient death, and the " + "sign on the curve indicates censoring (last follow-up time for patients who are still alive)

To validate the prognostic value of the 9 oxidative stress-related lncRNAs, the GEO validation cohorts were divided into high- and low-expression groups. Due to the small number of CRC samples in the GEO dataset, 5 lncRNAs (*AC034213.1, LINC01836, AP003555.1, AC083906.3, and AC009549.1*) were not expressed in the GEO dataset, so we grouped the sample based on the median expression of the remaining 4 lncRNAs. Unfortunately, the survival rate of CRC patients was not statistically significant between any of the high- and low-expression groups (*P* > 0.05) (Fig. 8). This is inconsistent with our previous results and may be related to the small sample size. Therefore, the exact role of these oxidative stress-related lncRNAs in CRC needs to be further investigated.

### Tumor mutation burden (TMB) analysis

**Fig. 9:**
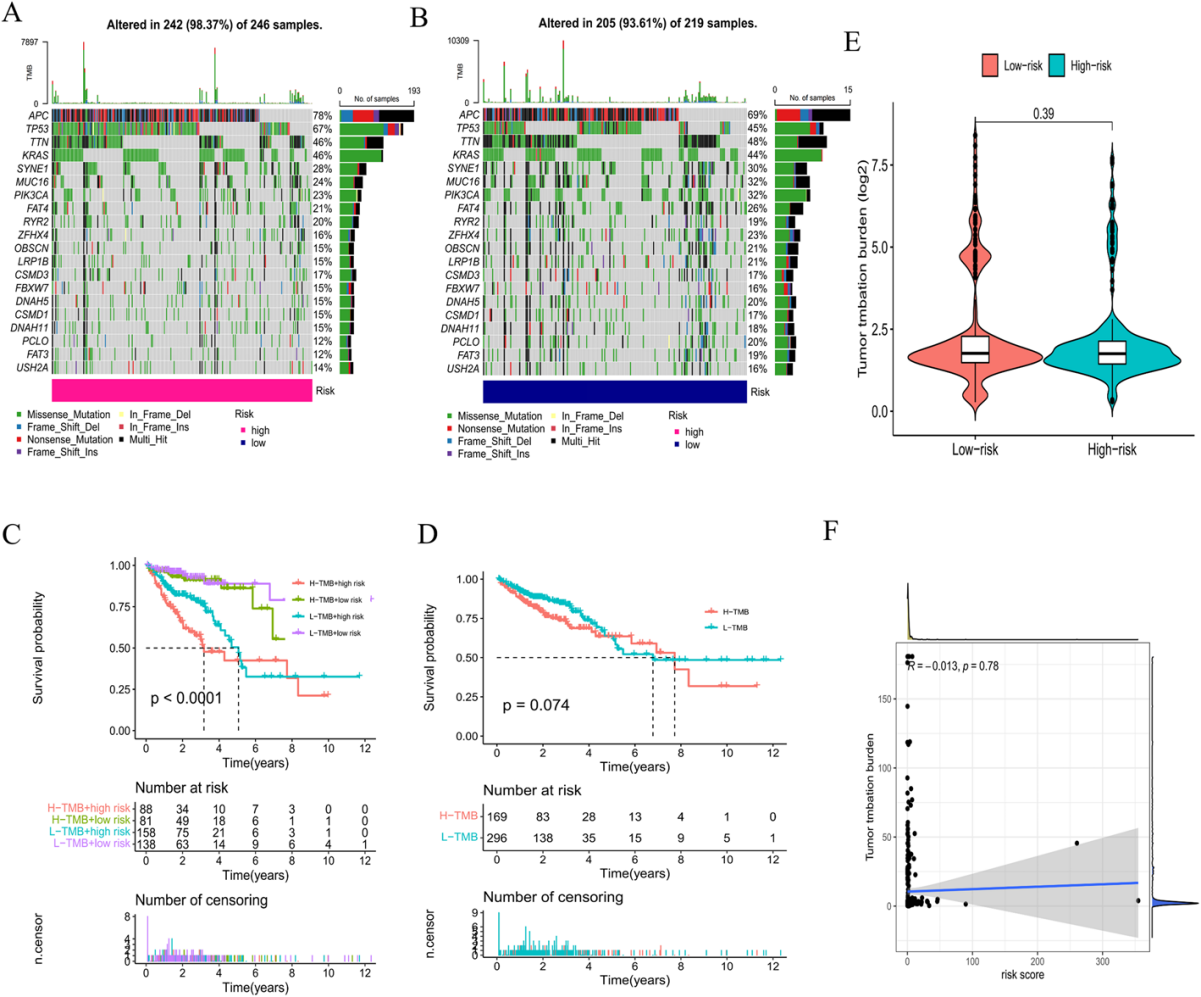
TMB analysis. The mutation rates in the high- (**A**) and low-risk groups (**B**). **C**–**D** Survival analysis of patients with different TMB levels. **E** Comparison of TMB for patients in the high- and low-risk groups. **F** Relevance analysis of TMB

It has been established that somatic mutations are a characteristic of CRC. Indeed, we found that APC, TP53, TTN, and KRAS had high mutation rates in both the low- and high-risk groups (TMB rate > 40%) (Fig. 9A, B). The OS of patients with CRC was significantly different among the high-TMB and high-risk, high-TMB and low-risk, low-TMB and high-risk, and low-TMB and low-risk groups (*P* < 0.001) but not significantly different between the low- and high-TMB groups (Fig. 9C, D). Unexpectedly, there was no difference in TMB between the high- and low-risk groups (Fig. 9E). Additionally, a weak negative correlation was observed between the risk score and TMB (R =  −  0.013) (Fig. 9F).

**Fig. 10:**
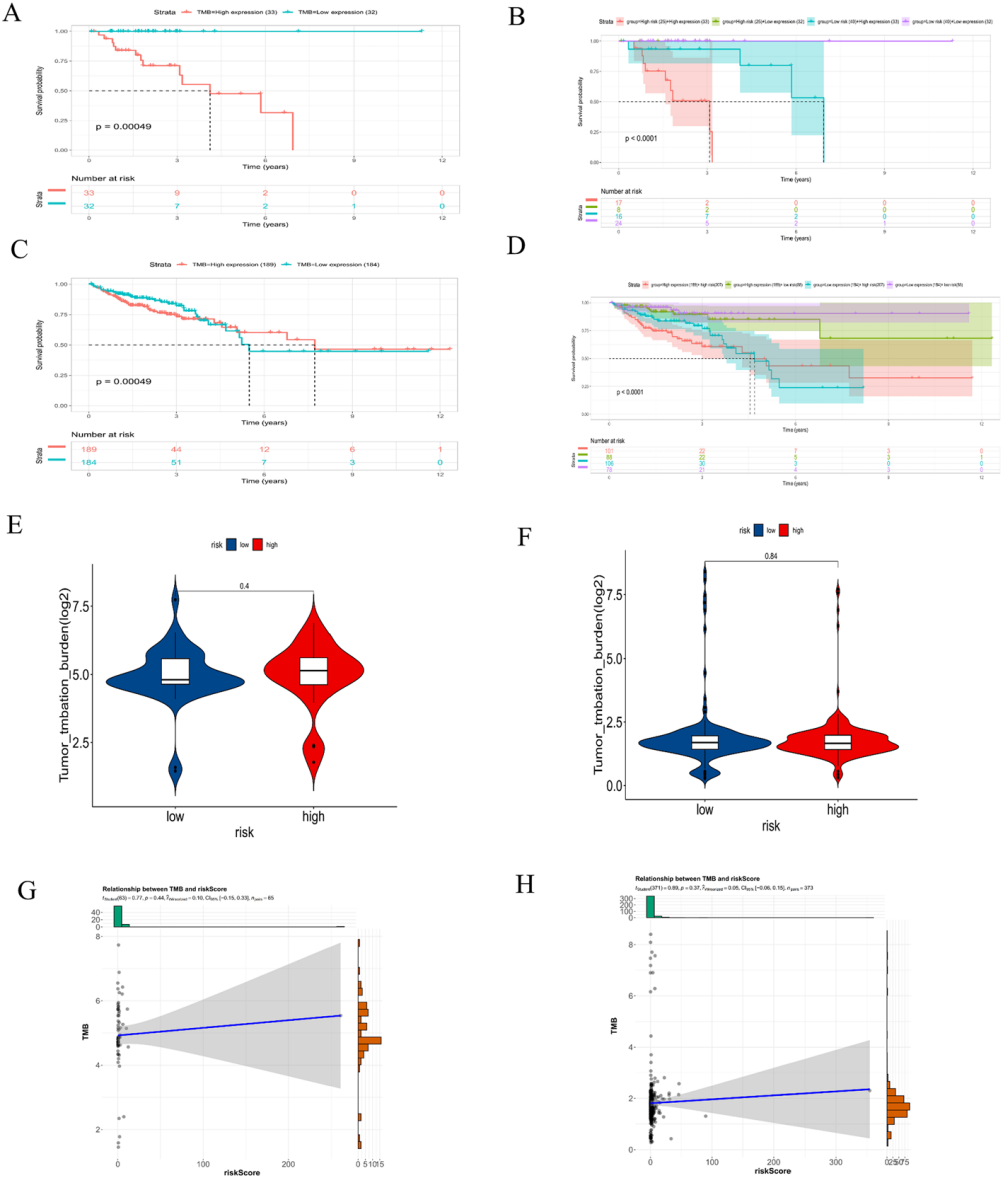
TMB analysis in the MSI-positive and MSI-negative groups. Survival analysis of patients with different TMB levels and different risk scores in the MSI-positive (**A**, **B**) and MSI-negative groups (**C**, **D**). Comparison of TMB in patients with high- and low-risk scores in the MSI-positive (**E**) and MSI-negative groups (**F**). Relevance analysis of TMB in the MSI-positive (**G**) and MSI-negative groups (**H**)

CRCs with microsatellite instability (MSI) have a significantly higher TMB than CRCs microsatellite stability (MSS). We regrouped the CRC samples according to the MSI score. Samples with MSI scores greater than 10 were classified as the positive group, and those that did not have MSI scores greater than 10 were classified as the negative group. Consistent with the results of previous studies, the low-TMB group showed a tendency toward prolonged survival (*P* < 0.001) (Fig. 10A, C). However, the subsequent stratified survival analysis showed that the risk score could distinguish the survival of patients with CRC in both the MSI-positive and MSI-negative subgroups and that the trend of the survival advantage in the MSI-positive group was reversed by the risk score (Fig. 10B, D). Unfortunately, there was no difference in TMB between the high- and low-risk groups in both the MSI-positive and MSI-negative groups (*P* > 0.05) (Fig. 10E, F). Inconsistently, a weak positive correlation was observed between the risk score and TMB in both the MSI-positive (R = 0.10) and MSI-negative groups (R = 0.05) (Fig. 10G, H).

### Gene set enrichment analysis (GSEA)

**Fig. 11:**
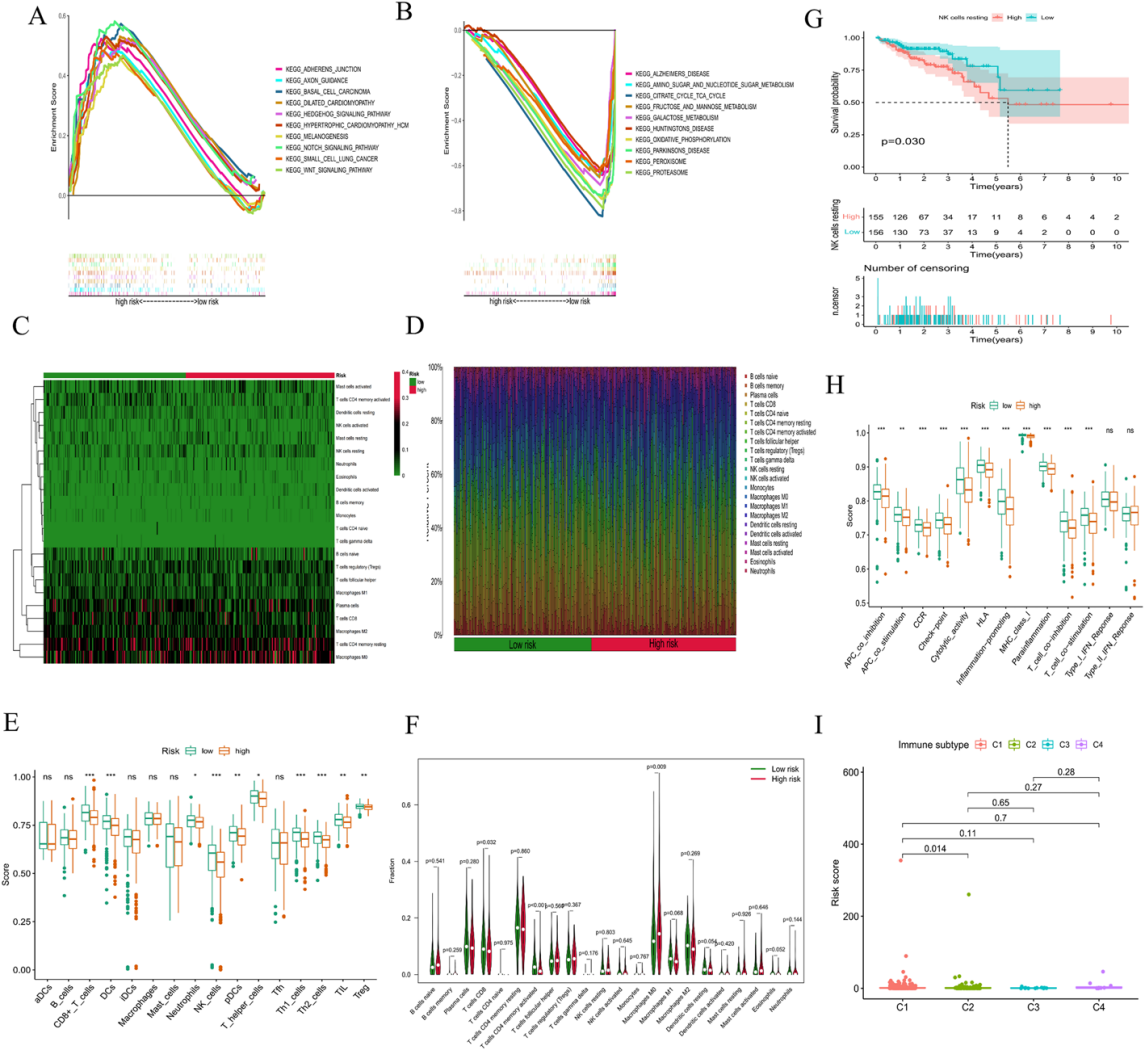
Investigation of immune cell infiltration and clinical treatment in different risk groups. GSEA of the top 10 pathways significantly enriched in the high-risk group (**A**) and low-risk group (**B**). **C**, **D** The immune cell heatmap of different risk groups. **E** The comparison of immune-related scores between the low- and high-risk groups. **F** The comparison of ratios of infiltrated immune cells between the low- and high-risk groups. **G** Survival curve of resting NK cells. **H** The comparison of immune functions between the low- and high-risk groups. **I** The comparison of immune subtypes between the low- and high-risk groups

To investigate differences in biological functions between the two subgroups, we analyzed the KEGG enriched pathways using GSEA. The top ten enriched pathways in the high- and low-risk groups were related to tumor invasion, metabolism, and immunity (Fig. 11A, B). More specifically, adherens junction, basal cell carcinoma, and the Wnt signaling pathway were primarily enriched in the high-risk group, while galactose metabolism, oxidative phosphorylation, and the proteasome were considerably enriched in the low-risk group. Therefore, we identified different enriched pathways in different subgroups by the prognostic model.

### Immune characteristics of the high- and low-risk subgroups

**Fig. 12:**
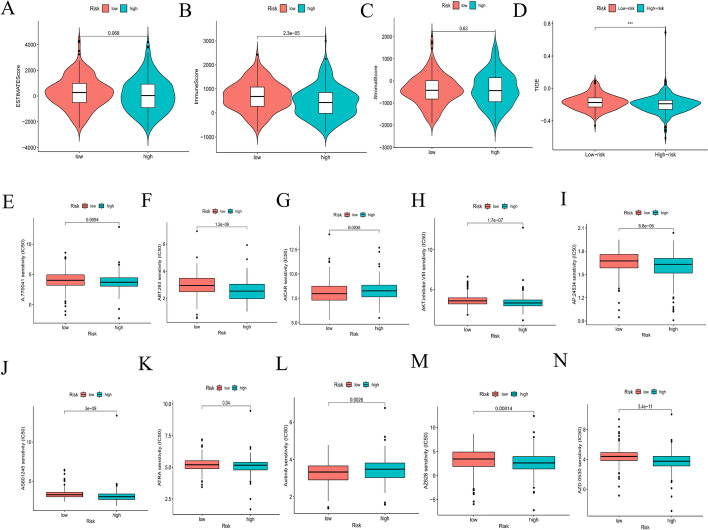
Investigation of immune cell infiltration and clinical treatment in different risk groups. **A**–**C** The comparison of immune-related scores between the low- and high-risk groups. **D** The comparison of the response to immunotherapy between the low- and high-risk groups. **E**‒**N** The immunotherapy prediction of different risk groups

The TME, which is the surrounding microenvironment of tumor cells, comprises immune cells, surrounding blood vessels, fibroblasts, extracellular stroma, and various signaling molecules. We first explored the relationship between the oxidative stress-related risk score and the TME. We found that the high-risk group presented higher immune cell infiltration than the low-risk group; however, our analysis showed that the low-risk group presented a higher ESTIMATE score and lower tumor purity than the high-risk group (Fig. 12A–C).

Heatmaps were constructed, as shown in Fig. 11C, D. The CIBERSORT algorithm was applied to estimate the ratios of infiltrated immune cells in the TMEs of the high- and low-risk groups. The results revealed that the ratio of infiltrated CD8^+^ T cells was notably higher in the low-risk group, while the ratios of infiltrated M0 macrophages and activated memory CD4^+^ T cells were markedly higher in the high-risk group. Similarly, ssGSEA showed that the ratios of infiltrated CD8^+^ T cells, dendritic cells, NK cells, TH1 cells, and TH2 cells were notably higher in the low-risk group (all P < 0.05) (Fig. 11E, F); moreover, the ratio of infiltrated resting NK cells was closely related to the OS of patients with CRC, which was significantly worse with an increased infiltration of resting NK cells (Fig. 11G). We also found that lower risk scores were more likely to be associated with immune function, such as APC co-inhibition, CCR (C–C chemokine receptor), check point, cytolytic activity, HLA, inflammation promotion, MHC class I, parainflammation, T-cell co-inhibition and T-cell co-stimulation (Fig. 11H). We also identified 4 immune subtypes in CRC patients (C1-C4) based on their ssGSEA scores. The risk scores were significantly different between the C1 and C2 subtypes (*P* < 0.05) (Fig. 11I). In summary, our results suggest that CRC patients in the low-risk group tend to have a more favorable (i.e., immune-activated) TME, while CRC patients in the high-risk group tend to have an immunosuppressive TME that can contribute to the immune escape of tumor cells and is related to a worse prognosis. Consistent with previous reports [23, 24], patients in the low-risk group also showed a better response to immunotherapy (Fig. 12D), indicating that our model could be used to select patients who are more likely to respond to ICT.

Chemotherapy drugs are the main treatment for patients with CRC. However, chemoresistance has been associated with a poor prognosis. Herein, we further predicted the chemotherapy response to common chemotherapy drugs in the two risk subgroups. The results showed that patients in the low-risk subgroup were more sensitive to AMPK inhibitors (AICAR) and tyrosine kinase inhibitors, while patients in the high-risk subgroup were more sensitive to Lck inhibitors (e.g., A.770041), Raf inhibitors (e.g., AZ628), and ATRA (Fig. 12E–N).

## Discussion

An increasing number of studies have confirmed that oxidative stress plays a crucial role in carcinogenesis and tumor treatment [25–27]. The construction of prognostic models based on public databases provides a more comprehensive clinical genetic prognostic value, and oxidative stress-based prognostic models are becoming a research hotspot for predicting the survival prognosis of different cancers [28–30]. However, the predictive value of oxidative stress-based prognostic models for the prognosis of CRC patients is still unknown and deserves further investigation. Using the transcriptome sequencing data, especially lncRNAs, and clinicopathological features of CRC obtained from TCGA, we identified and verified the 9-lncRNA prognostic signature related to OS in this study.

In the present study, 226 DEOSGs were identified based on the public TCGA database. Pathway enrichment analysis revealed that these DEOSGs were significantly correlated with the progression of several types of tumors, such as bladder, prostate, and hepatocellular carcinomas. Moreover, these DEOSGs were significantly enriched in several biological processes, including response to xenobiotic stimulus, response to oxidative stress, calcium ion homeostasis, and cellular divalent inorganic cation homeostasis, all of which have been reportedly correlated with tumorigenesis and progression [8, 31, 32]. Furthermore, we identified 2169 oxidative stress-related lncRNAs by coexpression analysis. More importantly, a total of 9 prognostic oxidative stress-related lncRNAs, i.e., *AC034213.1*, *AC008124.1*, *LINC01836*, *USP30-AS1*, *AP003555.1*, *AC083906.3*, *AC008494.3*, *AC009549.1*, and *AP006621.3*, were screened by univariate Cox and LASSO regression analyses. High expression of these 9 lncRNAs is related to good prognosis in patients with CRC. A previous study showed that Linc01836 may serve as a valuable noninvasive biomarker for the population screening, early detection, and clinical surveillance of CRC [33]. Other studies have found that AP0355.1 is closely associated with the poor prognosis of CRC and significantly correlated with OS, so it could be used as a promising biomarker for clinical outcome and therapeutic response predictions in colon cancer patients [34, 35]. In addition, the Sankey diagram showed that some of these lncRNAs were related to famous genes such as ATR and BRCA2. Some reports have shown that AC008124.1 is associated with ATR, and MMR promotes a DDR mediated by ATR, a key signaling kinase, in response to various types of DNA damage, including some encountered in widely used chemotherapy regimens [11]. In addition, some studies have shown that BRCA2 mutations are associated with CRC, but studies on how these mutations contribute to CRC development have shown conflicting results. For example, Gay-Bellile et al. [36] found that BRCA2 variants were implicated in familial CRC inheritance; however, one meta-analysis showed that there is an increased risk of CRC in BRCA1 but not BRCA2 mutation carriers [37–39]. Therefore, the conclusion from this study must be validated further in future large-scale studies.

The median risk score classified the patients into two groups, and K-M curve analysis showed that patients with high risk scores had a significantly worse prognosis; moreover, the stage factors were significantly related to the risk score. There was a tendency for a higher oxidative stress-related lncRNA-based risk score to be associated with a more advanced clinical stage. However, univariate and multivariate Cox analyses confirmed that the oxidative stress-related lncRNA-based risk score was not an independent predictor of OS. In addition, we built a nomogram to predict the OS at 1, 3 and 5 years as well as the risk of death. The performance of the nomogram was highly consistent with our prognostic model. Thus, our nomogram may provide simple but accurate prognostic predictions for CRC patients.

Using different analyses, we uncovered the underlying mechanisms of different risk groups identified by our prognostic model. GSEA indicated that the low-risk group contained a higher fraction of some immune-related cells and functions. According to previous studies, the tumor immune microenvironment can influence the prognosis of CRC patients [40, 41]. To determine whether the different prognoses of patients were related to tumor cell-mediated immunity, the infiltration levels of multiple immune cells in CRC patients were evaluated by different methods, including CIBERSORT and ssGSEA. We found that patients with high ratios of immune cell infiltration, including CD8^+^ T cells, dendritic cells, NK cells, TH1 cells, and TH2 cells, were mostly in the low-risk subgroup. It should also be noted that CD8^+^ T cells and NK cells favor antitumor activity in CRC and are associated with a better OS [42]. The TIDE module was used to estimate the immune function and rejection reaction of each patient. All of the above results indicate lower malignancy and potentially better immunotherapeutic effects in these patients [43, 44]. Thus, these results tentatively suggest that the poorer prognosis in the high-risk group may be due to dysregulation of antitumor immunity, and how oxidative stress affects the development of CRC through antitumor immunity still needs further study.

Moreover, the risk score was associated with TMB, suggesting that the poor prognosis of CRC patients in the high-risk group may be due to more mutated genes in this group. Increasing evidence suggests that patients with MSI-H are more sensitive to immunotherapy and can benefit from immunotherapy drugs [45]. As current immunotherapy is still in its infancy for CRC, patients with poor prognoses may benefit from immunotherapy due to their high TMB score with more mutated genes [46]. In the present study, the proportion of MSI-H patients was higher in the low-risk score group, and thus, the level of immune cell infiltration was subsequently upregulated in dMMR-MSI-H patients. Therefore, dMMR-MSI-H CRC patients may respond well to immune checkpoint blockade [47, 48]. CRC patients with MSI-H have both good and poor prognosis characteristics, and the specificity of MSI-H CRC determines the need for individualized treatment. The half maximal inhibitory concentration (IC50) was used to investigate the differences in sensitivity to common chemotherapeutic agents between the high- and low-risk groups.

In the current study, we established an oxidative stress-based prognostic model for predicting prognosis in CRC and revealed the relationship between oxidative stress-related lncRNAs and immune status. Nonetheless, there were still several limitations in our study. First, our prognostic model was constructed and validated using a TCGA cohort and requires other realistic prospective data to assess its future clinical predictive value. Second, further molecular biology experiments are needed to explore the relationship between oxidative stress and the prognostic features of CRC. Finally, the potential correlation between the risk score and antitumor immunity remains to be further investigated. Therefore, given the above limitations, the conclusions drawn from this study require more detailed validation.

## Conclusion

In summary, with a series of bioinformatic analyses, 9 oxidative stress-related lncRNAs were identified, which were related to the prognosis of patients with CRC. A prognostic model with powerful predictive ability was constructed. The relationships and underlying mechanisms among oxidative stress, lncRNAs, anti-immunity function, and CRC are worth further exploration.

## Methods

### Data acquisition and identification of differentially expressed oxidative stress-related genes (DEOSGs)

The RNA sequencing data of 568 CRC samples and 44 normal tissues, DNA mutation data of 582 CRC samples, and clinical information of 548 patients were downloaded from the TCGA database (https://portal.gdc.cancer.gov/). The gene expression profile matrixes of the CRC cohort from GSE192667 were downloaded from the GEO website (https://www.ncbi.nlm.nih.gov/geo/) for validation. To identify DEOSGs, we first curated a gene list containing 789 oxidative stress-related genes with a relevance score ≥ 7 on the GeneCards website (https://www.genecards.org) (Additional file 1), followed by transcriptional analysis with the limma package. The oxidative stress-related genes with a false discovery rate (FDR) < 0.05 and |log2-fold change (FC)|≥ 1 were identified as DEOSGs [49]. As a result, 226 DEOSGs were included for further analyses using the Wilcox.test.

### GO and KEGG pathway enrichment analyses

GO and KEGG pathway enrichment analyses were performed for the 226 DEOSGs, and the results were visualized using the clusterProfiler package (version 4.1.3) [50, 51]. Of note, GO analyses included biological process (BP), cellular component (CC), and molecular function (MF) analyses. A *P* value and adjusted *P* (q) value smaller than 0.05 were considered statistically significant.

### Selection of oxidative stress-related lncRNAs

A total of 16,773 lncRNAs were identified in the raw transcriptome data using Strawberry Perl and the limma package [52, 53]. Correlation analysis was performed between the expression of 226 DEOSGs (Additional file 2) and 16,773 lncRNAs. LncRNAs with Pearson correlation coefficients > 0.4 and *P* < 0.001 were selected. As a result, 2169 lncRNAs were identified as oxidative stress-related lncRNAs.

### Construction of the coexpression network

To better understand the relationship between lncRNAs and mRNAs, the lncRNA‒mRNA coexpression network was visualized by Cytoscape software (http://www.cytoscape.org/).

### Construction and validation of the prognostic model

To reduce statistical bias in the analysis, CRC patients with missing OS data or with short OS values (< 30 days) were excluded. Finally, we retrieved transcriptome, mutation, and clinical data for 506 patients. These patients were randomly grouped into the training and testing groups at a ratio of 1:1 using Strawberry Perl and the caret package. All oxidative stress-related lncRNAs were subjected to univariate Cox regression analyses to explore the relationship between lncRNA expression and patient OS by the survival package. LncRNAs with a *P* < 0.05 were identified as prognostic oxidative stress-related lncRNAs. Subsequently, these candidate lncRNAs were integrated into least absolute shrinkage and selection operator (LASSO) regression to construct a prognostic model [54]. The risk score of each patient was calculated with the following formula:

$${\text{Risk score}} = \sum\limits_{{{\text{n}} = 1}}^{{\text{k}}} {\exp {\text{r}} - \left( {\ln {\text{cRNA}}} \right)~*~{\upbeta }{\text{i}}}$$

where expr(lncRNA) represents the short form of the expression of lncRNAs correlated with survival and βi represents the regression coefficient.

The CRC patients were grouped into low- and high-risk subgroups based on the median risk score [52, 55]. The Kaplan‒Meier method and log-rank test using the R Bioconductor survival package were further conducted to compare OS [56, 57]. Scatter plots were used to display the survival status of CRC patients in the low- and high-risk groups. Utilizing the pheatmap package, a heatmap was constructed to show the differential expression of 9 lncRNAs between the low- and high-risk groups. The effectiveness and accuracy of the model were further verified using the survival and timeROC packages [58]. Univariate and multivariate Cox regression analyses were also performed to evaluate the relationship between clinical characteristics and the risk score. Additionally, a concordance index (C-index) curve was constructed to evaluate the prediction accuracy of the model in CRC patients using the pec package [59]. Risk score, age, sex, and tumor stage were used to construct a nomogram for predicting 1-, 2-, and 3-year OS using the rms package. Correction curves based on the Hosmer Lemeshow test showed that the predicted prognosis was in good agreement with the actual outcome [60, 61]. Finally, PCA and t-SNE were performed by the Rtsne and scaterplot3d packages to determine whether two subgroups could be distinguished by these two metrics [62, 63].

### Evaluation of the prognostic value of 9 oxidative stress-related lncRNAs in an independent GEO validation cohort

The CRC patients in GEO were grouped into low and high expression subgroups based on the median level of the lncRNAs [64]. The Kaplan‒Meier method using the R Bioconductor survival package was further conducted to compare the OS between the two subgroups, and then the survival and survminer packages were used to plot the survival curves.

### TMB analysis

The DNA mutation data of CRC patients were downloaded from the TCGA database. The mutation rates in the high- and low-risk groups were visualized using the maftools and Rhtslib packages. Differences regarding TMB and OS in the high- and low-risk groups were analyzed using the limma, ggpubr, and survivor packages [65]. A relevance analysis between the risk scores and TMBs was performed using the limma, ggpubr, and ggExtra packages.

We further downloaded the reference list of MSI scores from the cBioPortal database (https://www.cbioportal.org/) (Additional file 3) [66]. Samples with MSI scores greater than 10 were classified as the positive group, and those with MSI scores not greater than 10 were classified as the negative group. In the MSI-positive and MSI-negative groups, the ggplot2, survivor and ggstatsplot packages were used to perform difference analysis, survival analysis and correlation analysis between TMB and the risk score [67, 68].

### GSEA

GSEA (https://www.gsea-msigdb.org/gsea/login.jsp) was performed using the “kegg.v7.4.symbols.gmt” gene set to identify significantly enriched pathways between the low- and high-risk groups. Pathways with *P* < 0.05 and FDR < 0.25 were regarded as significantly enriched pathways [29].

### Analyses of TME and immune cell infiltration

The ESTIMATE scores, immune scores, and stromal scores of CRC samples were estimated based on transcriptional data using the “estimation of stromal and immune cells in malignant tumors using expression data” (ESTIMATE) method [69]. Tumor purity was inferred based on the ggpubr package. Moreover, the abundances of 22 tumor-infiltrating immune cells were estimated using the “cell-type identification by estimating relative subsets of RNA transcripts” (CIBERSORT) algorithm [70]. Utilizing the limma, ggpubr, pheatmap and vioplot packages, a heatmap was constructed to show the differential expression of immune cells between the low- and high-risk groups and the relative expression of immune cells in each sample. Immune cell infiltration was also estimated using single-sample GSEA (ssGSEA) [71, 72]. To assess the effectiveness of our model in predicting immunotherapy response, the online tool, Tumor Immune Dysfunction and Exclusion (TIDE) (http://tide.dfci.harvard.edu/), was used to estimate patient response to immune checkpoint therapy.

### Exploration of the model in clinical treatment

The clinical responses of CRC patients to different chemotherapy drugs were predicted using the pRRophetic package. We compared the half-maximal inhibitory concentration (IC50) between the high- and low-subgroups [73].

## Supplementary Information

**Additional file 1**. The List of 789 oxidative stress-related genes.

**Additional file 2**. The List of 226 differentially expressed oxidative stress-related genes.

**Additional file 3**. The reference list of MSI scores.

## Data Availability

The datasets used for analyses in the current study are available from the corresponding author upon reasonable request.

## Abbreviations

AMPK: Adenosine 5′-monophosphate (AMP)-activated protein kinase
Akt: (PKB) protein kinase B
APC: Antigen-presenting cell
ATR: ATM and Rad3-related
ATRA: All-trans retinoic acid
ATM: Ataxia telangiectasia mutated
AUC: Area under the ROC curve
BP: Biological process
CAR-T: Chimeric antigen receptor T-cell immunotherapy
CC: Cellular component
CCR: C–C chemokine receptor
CIBERSORT: Cell-type identification by estimating relative subsets of RNA transcripts
CRC: Colorectal cancer
DDR: DNA damage response
DEOSGs: Differentially expressed oxidative stress-related genes
dMMR-MSI-H: Mismatch repair deficient high microsatellite instability
ESTIMATE: Estimation of stromal and immune cells in malignant tumors using expression data
FDR: False discovery rate
GEO: Gene expression omnibus
GO: Gene ontology
GSEA: Gene set enrichment analysis
HLA: Human leukocyte antigen
IC50: Half-maximal inhibitory concentration
ICT: Immune-checkpoint therapy
JNK: C-junN-terminal kinase
KEGG: Kyoto encyclopedia of genes and genomes
LASSO: Least absolute shrinkage and selection operator
Lck: Lymphocyte-specific protein tyrosine kinase
MDSCs: Myeloid-derived suppressor cells
MF: Molecular function
MHC: Major histocompatibility complex
MMR: Mismatch repair
MSI: Microsatellite instability
MSS: Microsatellite stability
multi-Cox: Multivariate Cox regression
NF‐κB: Nuclear factor κB
OS: Overall survival
PCA: Principal component analysis
PI3K: Phosphatidylinositol 3‐kinase
ROC: Receiver operating characteristic
ROS: Reactive oxygen species
RTK: Receptor tyrosine kinase
ssGSEA: Single-sample gene set enrichment analysis
TCGA: The cancer genome atlas
TIDE: Tumor immune dysfunction and exclusion
TMB: Tumor mutation burden
TME: Tumor environment
Tregs: Regulatory T cells
t-SNE: T-distributed stochastic neighbor embedding
